# YMGD: A yemeni music genres database with audio recordings, mel-spectrograms, and metadata

**DOI:** 10.1016/j.dib.2026.112859

**Published:** 2026-05-17

**Authors:** Eiad AL-Mekhlafi, Moeen AL-Makhlafi, Saher Qaida, Ahmed Asqeina, Ahmed Alawadhia, Nawaf Q. Othman

**Affiliations:** a Department of Computer Science and Information Technology, IBB University, IBB, Yemen; b College of Science and Engineering, National University, IBB, Yemen; c School of Information Engineering, Wuhan College, Wuhan 430212, Wuhan, Hubei, China; d School of Information Engineering, Xian Fanyi University, Xi’an 710105, Shaanxi, China

**Keywords:** Data science, Music genres classification, Spectrogram analysis, Dataset benchmarking, Yemeni music dataset

## Abstract

The Yemeni Music Genres Dataset (YMGD) is the first curated benchmark for Arabic Yemeni musical traditions, comprising a balanced collection of five genres, Sana'ani, Hadhrami, Tihami, Lahji, and Adeni, with 230 audio recordings per class, each standardized to 30 s. In addition to the audio content, the dataset includes comprehensive metadata for each track, including artist name, genre label, and title, enabling structured analysis and reproducibility. The dataset was manually collected from publicly available sources, primarily YouTube, and subsequently annotated by domain experts in Yemeni music to ensure high labeling fidelity. Inter-annotator agreement was quantified using Fleiss’ Kappa, yielding a score of 0.85, indicating strong consistency and reliability in the annotation process. This dataset provides a robust foundation for a wide range of research applications, including music information retrieval, the development and evaluation of machine learning models for genre classification, recommendation systems, and computational cultural analysis. By combining expert validation with balanced representation and standardized preprocessing, it establishes a high-quality benchmark resource for both research and educational use in low-resource musical domains. The dataset will be accessible at the following link: https://doi.org/10.5281/zenodo.19543208.

Specifications Table

**Table:** 

Subject	Computer Sciences
Specific subject area	Songs Genre Classification, Music Information Retrieval.
Type of data	Audio (WAV files)
Data collection	Data were manually collected from YouTube, with assistance from expert judges on Yemeni songs. Audio files were processed and saved in 30-second clips.
Data source location	City: IBB Country: Yemen
Data accessibility	Repository name: YMGD: A Yemeni Music Genres Database Direct URL to data: https://doi.org/10.5281/zenodo.19543208.

## 1 Value of the Data

This dataset constitutes the first publicly available collection dedicated to Yemeni music genres, offering a structured and comprehensive foundation for the study of regional musical styles. Its introduction addresses a critical gap in culturally specific datasets within music information retrieval and related computational domains.

The dataset is designed to support a broad spectrum of research communities, including scholars in music information retrieval, machine learning, and deep learning for classification tasks, as well as cultural and ethnomusicological studies. By enabling cross-disciplinary investigation, it facilitates both technical advancements and cultural understanding.

A key strength of the dataset is its balanced composition, with 230 audio tracks per genre. This uniform distribution mitigates class imbalance issues and supports robust model training and evaluation, thereby enabling reliable and reproducible genre classification.

Beyond its primary use, the dataset offers substantial reusability across multiple research directions. It can support comparative analyses between Yemeni music and other traditional or contemporary genres, contribute to deeper insights into regional musical structures, and serve as a foundation for developing educational and intelligent systems, including automated genre classification and music recommendation models.

## 2 Background

Music classification has emerged as a rapidly evolving and critically important research area within music information retrieval, driven by advances in neural networks and deep learning techniques [[1], [2], [3], [4]]. Despite these developments, the task remains challenging due to the complexity and diversity of musical structures. Yemeni music, in particular, is characterized by significant regional diversity, with Sana'ani music recognized by UNESCO as an intangible cultural heritage [5]. A fundamental challenge in this domain is the scarcity of high-quality, well-annotated datasets. While Arabic music content is increasingly available online, it is often poorly structured and inconsistently labeled across genres [6], with Yemeni music being even more underrepresented. This lack of curated data significantly limits the development and evaluation of robust classification models.

Existing studies on Arabic music classification have demonstrated promising results using relatively small datasets. For instance, Elshaarawy and Saeed [7] reported an accuracy of 81.4% in composer-based classification, while ElAlam and Tobar [8] achieved 99.2% accuracy using support vector machines. Furthermore, substantial progress has been made in the analysis of Arabic [9,10], Saudi [11], Moroccan [12], and Egyptian music [[13], [14], [15], [16]]. However, research specifically focused on Yemeni music remains limited. To address this gap, this paper introduces a curated dataset of Yemeni music genres, designed to provide high-quality, well-structured data across five principal genres: Sana'ani, Hadhrami, Tihami, Adeni, and Lahji.

## 3 Data Description

**Fig. 1:**
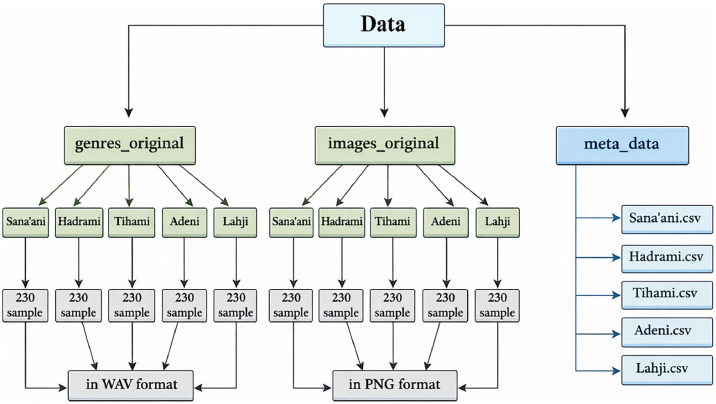
YMGD dataset structure.

The dataset is organized into multiple components, including raw audio files, spectrogram image representations, and structured metadata, as illustrated in Fig. 1.

1- Genres_Original folder:
  This directory contains the primary audio data, comprising 1150 clips distributed across five Yemeni music genres. Each genre includes 230 audio files, with all recordings standardized to a duration of 30 s. The files are stored in WAV format, with a sampling rate of 48 kHz and a bit rate of 360 kbps, ensuring consistent quality for audio analysis and machine learning tasks.
2- Images_original folder:
  This folder provides visual representations of the audio signals in the form of Mel-spectrogram images. These time–frequency representations enable the application of convolutional neural networks (CNNs) by transforming audio signals into a format suitable for image-based classification.
3- Meta_data folder:
  This directory contains five CSV files, each corresponding to one genre. These files include structured metadata for each audio clip, such as: Song number, Sample number, Song title, Artist name, and Genre label. This metadata facilitates dataset traceability, organization, and reproducibility in experimental settings.

**Fig. 2:**
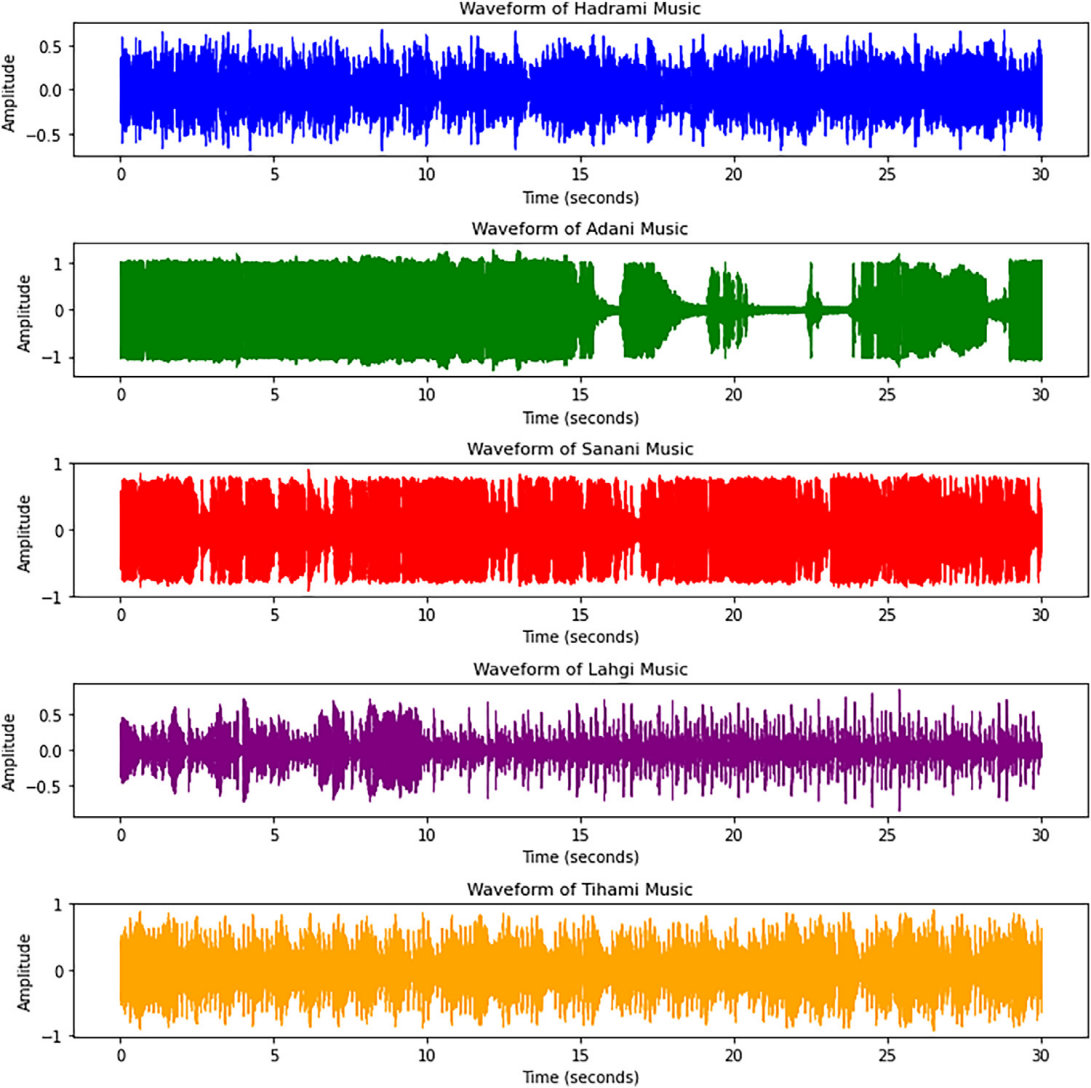
Waveform representations of five YMGD.

Fig. 2 presents waveform visualizations of five Yemeni music genres, Hadhrami, Adeni, Sana’ani, Lahji, and Tihami, over a 30-second duration. Each subplot illustrates the amplitude variations of the audio signal over time, highlighting distinct temporal and dynamic characteristics across genres. Notable differences can be observed in signal intensity, density, and patterns of variation; for instance, some genres exhibit more continuous, dense waveforms, while others show intermittent fluctuations and varying energy levels. These variations reflect the inherent acoustic and rhythmic diversity of Yemeni musical styles, providing a meaningful basis for distinguishing genres in audio analysis and machine learning tasks.

## 4 File Labeling

**Fig. 3:**
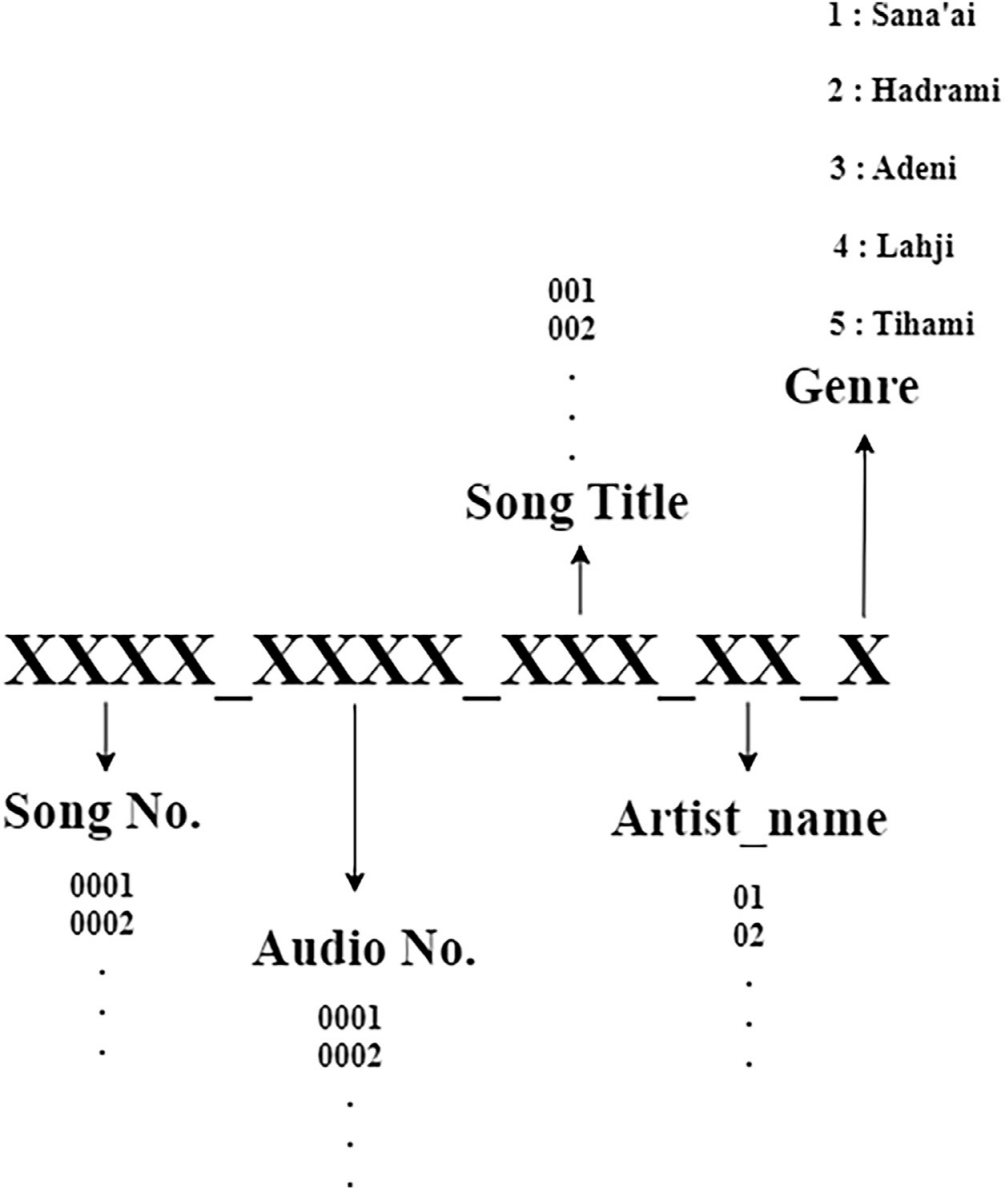
Data Labeling structure.

Manual annotation was performed by domain experts in Yemeni music to assign each audio track to its corresponding genre. Each track was labelled using a structured identifier composed of five elements separated by underscores, encoding information such as genre and dataset indexing (as illustrated in Fig. 3. This systematic labeling scheme ensures both consistency and traceability across the dataset.

## 5 Experimental Design, Materials and Methods

**Fig. 4:**
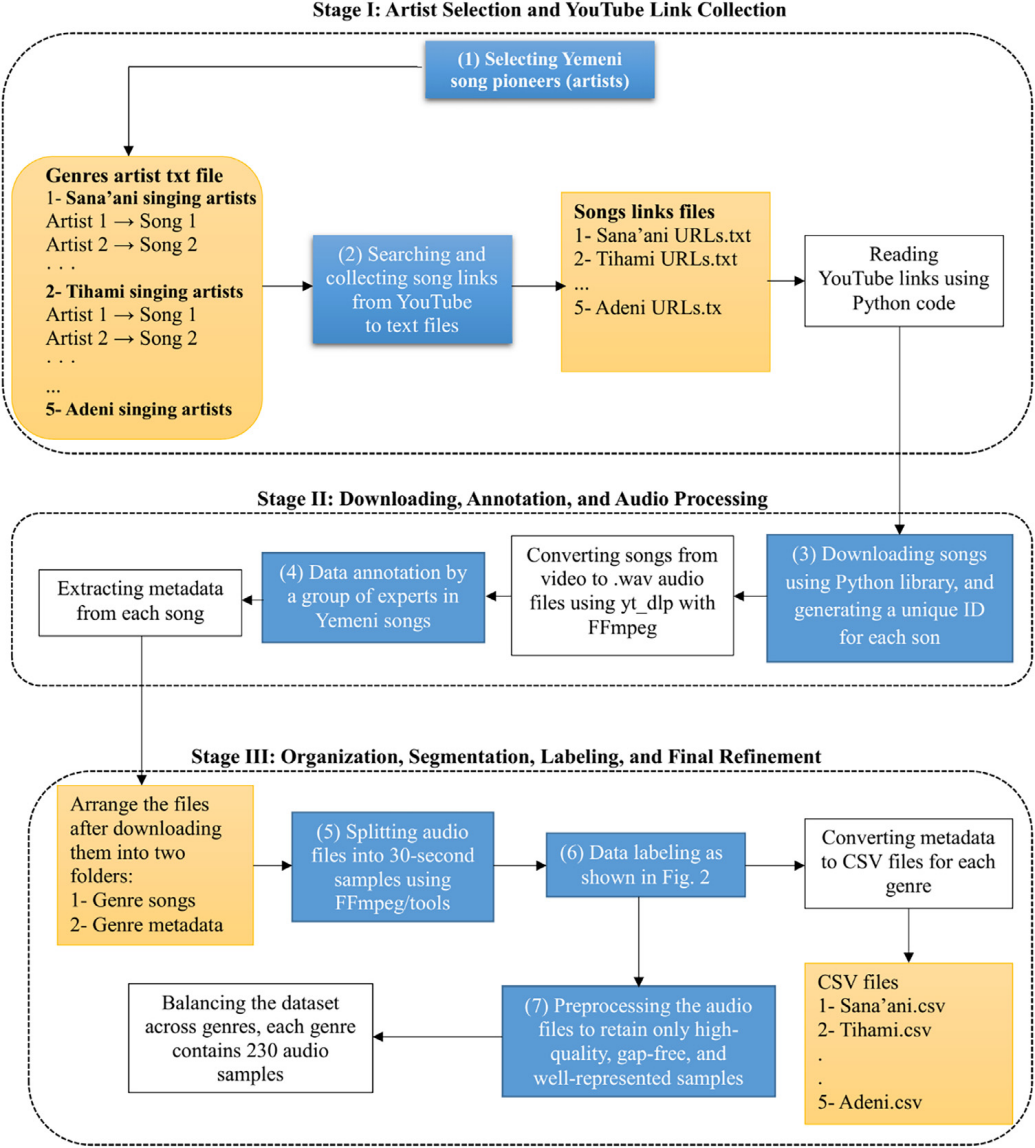
Data flow diagram illustrating the dataset collection, annotation, and processing pipeline.

The Yemeni Songs Dataset is a structured, comprehensive repository of Yemeni music, encompassing five principal genres: Sana'ani, Hadhrami, Lahji, Tihami, and Adeni. The dataset is carefully curated to support advanced audio analysis, machine learning applications, and computational studies of Yemeni musical traditions. The data collection and organization process was implemented through a systematic, multi-stage pipeline using Python. This experimental workflow enabled automated data retrieval, processing, and structuring, ensuring consistency and reproducibility. The overall data collection procedure is summarized in the data flow diagram presented in Fig. 4.

1. **Selection and Categorization of Artists:** Initially, prominent Yemeni artists were identified and systematically categorized into five groups corresponding to the defined genres. For each artist, a curated list of their most representative songs was compiled and stored in dedicated text files, as illustrated in Fig. 3. This step established a comprehensive and genre-balanced foundation for subsequent data collection.
2. **Link Collection and Organization:** Using the compiled song lists, the corresponding YouTube links were retrieved and organized into five genre-specific text files. These files were processed programmatically using Python to ensure efficient and accurate handling of the data sources.
3. **Data Acquisition and Format Standardization**: Audio data were downloaded using the Pytube library. Each track was assigned a unique identifier to ensure traceability. The downloaded videos were then converted into WAV format, preserving audio quality while standardizing the format for downstream processing.
4. **Expert Annotation and Data Structuring:** The dataset was annotated by a panel of Yemeni music experts, who assigned genre labels based on domain-specific knowledge of musical characteristics. This expert-driven process ensured high labeling accuracy and reliability. Following annotation, the data were organized into structured directories, separating raw audio files and genre-specific metadata, as illustrated in Fig. 1.
5. **Audio Segmentation:** Each audio file was segmented into 30-second fixed-length clips. Each segment was assigned a sub-identifier linked to the original track, preserving traceability and enabling fine-grained analysis.
6. **Labeling and Metadata Construction:** All audio segments were systematically labeled using a structured naming convention (see Fig. 3). Metadata for each genre were compiled into CSV files, facilitating efficient data management, analysis, and integration into machine learning pipelines.
7. **Data Cleaning and Balancing:** In the final stage, the dataset underwent rigorous preprocessing. Low-quality or incomplete audio samples were removed to ensure data integrity. To maintain class balance and avoid bias, each genre was limited to 230 audio files, resulting in a uniformly distributed dataset suitable for reliable model training and evaluation.

## 6 Data Annotation and Validation

Five independent annotators with extensive expertise in Yemeni music genres participated in the labeling process. To ensure consistency and reliability, all annotators were provided with detailed guidelines and a standardized annotation protocol. Each annotator independently reviewed every audio sample and assigned it to one of the five predefined genres: Sana'ani, Hadhrami, Lahji, Tihami, or Adeni.

In cases of disagreement among annotators, a structured resolution protocol was employed to ensure label consistency and reliability. Initially, annotators engaged in discussion and consultation to examine the basis of their differing judgments and to reach a consensus. For particularly challenging cases, additional expert input was solicited to resolve ambiguity. If consensus could not be achieved through discussion, a majority voting scheme was used, in which the final label was determined by the agreement of the majority of annotators. In instances where a track remained highly ambiguous or could not be confidently assigned to a specific genre, annotators were instructed to leave it unannotated to avoid introducing noise into the dataset. This multi-stage validation process ensured the integrity, accuracy, and reliability of the final annotations.

**Fig. 5:**
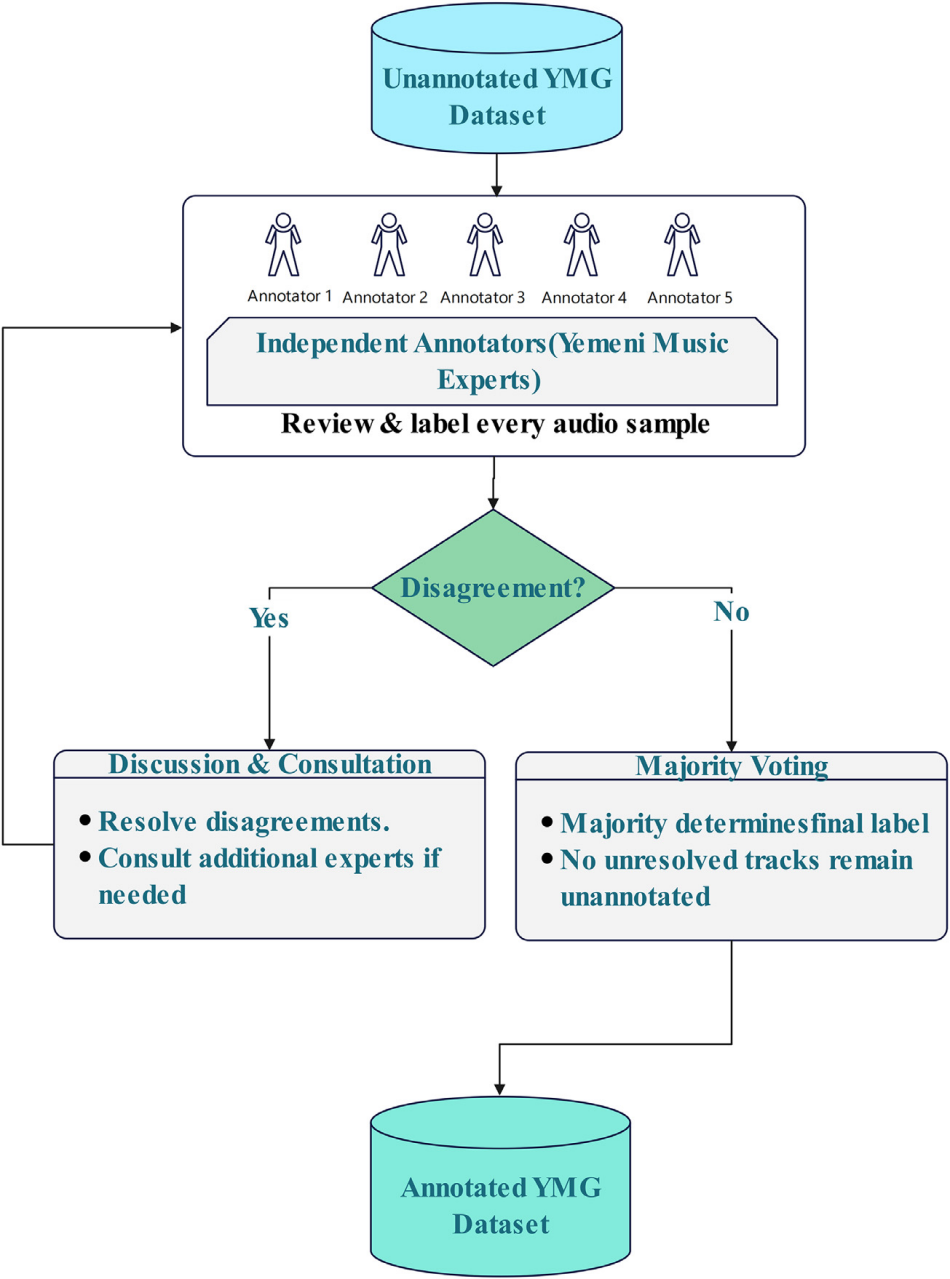
**YMGD** dataset annotation process.

Fig. 5. presents a visual overview of the data annotation workflow for the Yemeni song genres dataset, illustrating each stage from the initial unannotated data to the final validated labels. The adoption of a structured and multi-stage annotation process ensures high precision and consistency in the assigned genre labels. To quantitatively assess the reliability of the annotations, inter-annotator agreement was evaluated using Fleiss’ Kappa [5], a widely used statistical measure for categorical classification tasks. This evaluation provides a robust indication of the consistency and validity of the labeling process.

Fleiss’ Kappa is computed using the following formulation:

$$k=\frac{p_{o}-p_{e}}{1-p_{e}}$$

Here, *p_o_* denotes the observed agreement among the annotators, while *p_e_* represents the expected agreement due to chance. A value of *κ=1* indicates perfect agreement among all raters.

The evaluation yielded a Fleiss’ Kappa score of 0.85, indicating a high level of inter-annotator agreement and strong consistency in the assigned labels. This result demonstrates the effectiveness and rigor of the annotation process, confirming the dataset's reliability. The substantial agreement among annotators validates the adopted annotation protocol and supports the dataset's overall quality and robustness. Consequently, the dataset provides a dependable foundation for subsequent analysis and machine learning applications.

## Limitations

As the first dataset of its kind on Yemeni music, it encountered several challenges during its construction. One of the primary difficulties stemmed from the high similarity between different Yemeni musical genres, which complicated the annotation process and increased the potential for ambiguity in genre assignment. Additionally, the scarcity of high-quality, well-documented audio data, coupled with the limited availability of reliable data sources, posed significant constraints on data collection. Furthermore, the annotation process required substantial time and effort, as expert annotators needed to carefully evaluate each audio sample to ensure accurate classification. The inherent similarity among genres further prolonged this process, necessitating meticulous review to maintain labeling precision and consistency.

## Ethics Statement

This study was conducted in accordance with established ethical standards for scientific research, with particular attention to data usage, intellectual property, and participant rights. The research protocol was reviewed and approved by the IBB University Research Ethics Committee, confirming compliance with relevant ethical guidelines.

During dataset construction, several key ethical considerations were strictly observed:

- Compliance with platform policies: All data collection procedures adhered to YouTube’s Terms of Service, ensuring respect for content ownership and creators’ rights.
- Data privacy and integrity: Metadata associated with each audio sample was handled responsibly to preserve data integrity and prevent misuse of artist-related information.
- Licensing and data governance: Appropriate licensing terms were defined for the dataset to regulate its distribution and usage, promoting responsible and transparent reuse in research contexts.

The research team affirms its full commitment to ensuring the integrity of the research and respecting the rights of participants in accordance with international standards of scientific ethics.

## CRediT Author Statement

**Eiad AL-Mekhlafi:** Conceptualization, methodology, writing. **Moeen AL-Makhlafi:** Conceptualization, supervision, Review and Editing. **Saher Qaida:** Conceptualization, methodology, data Construction, writing, original Draft. **Ahmed Asqeina:** Conceptualization, methodology, data Construction, writing, original Draft. **Ahmed Alawadhia:** Conceptualization, data Construction. **Nawaf Q. Othman:** Writing, Review and Editing.

## Data Availability

zenodoYMGD: A Yemeni Music Genres Database (Original data)
